# Severity of Adverse Effects of Sinovac COVID-19 Vaccine in Postmenopausal Women: A Multicenter Experience From Pakistan

**DOI:** 10.7759/cureus.46682

**Published:** 2023-10-08

**Authors:** Asma Safdar Syed, Shazia Sultana, Amna Begum, Khasheaa Nadeem, Jamal Ara, Saira Hassan Askarey, Ahsan Ali Siddiqui, Adnan Anwar, Atif A Hashmi

**Affiliations:** 1 Obstetrics and Gynecology, Chiniot General Hospital, Karachi, PAK; 2 Obstetrics and Gynecology, Ziauddin University, Karachi, PAK; 3 Obstetrics and Gynecology, Abbasi Shaheed Hospital, Karachi, PAK; 4 Internal Medicine, Karachi Medical and Dental College, Karachi, PAK; 5 Internal Medicine, Abbasi Shaheed Hospital, Karachi, PAK; 6 Physiology, Dow University of Health Sciences, Karachi, PAK; 7 Family Medicine, Tumair General Hospital, Riyadh, SAU; 8 Physiology, Hamdard College of Medicine and Dentistry, Karachi, PAK; 9 Internal Medicine, Essa General Hospital, Karachi, PAK; 10 Pathology, Liaquat National Hospital and Medical College, Karachi, PAK

**Keywords:** swelling, pain, fever, post-menopausal women, vaccine, covid-19, sinovac

## Abstract

Introduction

The most significant element in halting the coronavirus disease 2019 (COVID-19) epidemic was the availability of reliable and efficient vaccines. Vaccine acceptability is influenced by many factors, including perceptions of the vaccine’s safety and side effects. Adverse reactions to vaccines can vary with regard to the type, although they are frequently mild, localized, temporary, and self-limiting. Therefore, this study aimed to assess the prevalence of side effects experienced by postmenopausal women after receiving the Sinovac vaccine.

Methods

This multicenter, prospective cross-sectional study was carried out at multiple centers in Karachi, Pakistan. In this study, the non-probability sampling method was used. The study continued from August 1, 2022, to January 31, 2023, for six months. The study comprised 600 postmenopausal women over the age of 50 years who received two doses of Sinovac COVID-19 vaccination. Demographic parameters such as gender, the existence of comorbidities, and local and systemic side effects in postmenopausal women were documented as frequencies and percentages. Age, weight, and duration of comorbidities are expressed as means and standard deviations.

Results

The study findings showed that the mean age of study participants was 63.93 ± 8.24 years. There were related comorbidities with hypertension and diabetes mellitus in 181 (30.2%) and 40 (6.7%) women, respectively. Fever was the most often reported side effect, with 349 (58.2%) participants reporting it and 198 (56.7%) participants reporting it as mild. After the second dose, 234 (39.5%) participants reported fever as their most frequent adverse effect, and 158 (67.5%) of them reported it was mild.

Conclusion

This study concluded that the most commonly reported side effects among postmenopausal women were fever, pain, and swelling at the injection site after getting either dose of Sinovac vaccine. These overall side effects were generally mild to moderate in intensity, not life-threatening, and did not require hospitalization, although fever was reported in severe intensity in some cases, particularly after the first dose.

## Introduction

The severe acute respiratory syndrome coronavirus 2 (SARS-CoV-2) epidemic that occurred in Wuhan, China, in December 2019 created a terrible global crisis. Coronavirus disease 2019 (COVID-19) has no known treatment. For the epidemic to be controlled, vaccination was essential. Worldwide, vaccinations continue to prevent millions of fatalities each year by providing either complete immunity from the illness or alleviation from its manifestations. The first COVID-19 vaccine recipient swiftly started clinical assessment on March 16, 2020 [1]. The emergency use of the CoronaVac vaccine for medical experts, older people (over 65 years), and those with coexisting illnesses was approved by Turkey on January 14, 2021. Primarily, two doses were administered at four-week intervals [2].

Since the first COVID-19 vaccines received approval for recommendation in the emergency endemic crisis in December 2020, millions of vaccinations have been administered worldwide [3]. There are three primary types of COVID-19 vaccinations that are currently being used in different parts of the world. The first category includes mRNA-based vaccinations such as Moderna (mRNA-1273) and Pfizer-BioNTech (BNT162b2). Vaccines against inactivated whole viruses (Sinopharm/BIBP-CorV and Sinovac Biotech) and recombinant adenoviral vectors (Sputnik V, Johnson & Johnson/Janssen/Ad26.COV2.S, and Oxford-AstraZeneca/ChAdOx1 ncov-19) are included in the second and third classes, respectively. There is evidence of both moderate negative consequences, including weariness, headache, and myalgia, as well as more serious side effects after administration [4,5].

In several countries, the Sinovac vaccine (a Chinese inactivated virus vaccine) is one of the most frequently used vaccinations. It is predicted that the efficacy rates are 83.5% and 95% for Sinovac and Pfizer vaccines, respectively [6,7]. The Sinovac vaccine has one of the highest coverage rates of any other vaccine, and investigation of potential side effects has been conducted to increase its efficacy. On the basis of many clinical investigations of the COVID-19 vaccine, fever or allergic responses, including itchiness and inflammation, were among the less severe reactions initiated by COVID-19 vaccines [8-10], whereas injection site pain and swelling were commonly observed adverse reactions with a recovery rate of 48 h following vaccination. Furthermore, moderate side effects of the Sinovac vaccine included tiredness, muscular pain, and diarrhea, which persisted for two days. In contrast to the other COVID-19 vaccines, CoronaVac/Sinovac participants had fewer fever episodes. For individuals 18 years of age and older, the Sinovac vaccine is advised [10].

Vaccine hesitancy, which is exacerbated by misleading information and prejudices about the effectiveness and safety of vaccines, is a serious public health concern [11]. A few of the factors that can influence vaccine adoption are awareness of the vaccine, perceptions regarding its negative effects, opinions about vaccination, observed vulnerability to illness, societal implications, and increasing vaccine information [12]. Healthcare professionals from Western Arab countries (Morocco, Egypt, Tunisia, and Algeria) have the highest rates of vaccination resistance, according to a comprehensive worldwide survey. Fear of side effects was the most frequently stated reason for vaccine avoidance [13].

COVID-19 vaccination faces resistance, particularly in older and postmenopausal women [11]. Our study examined the relationship between the coronavirus vaccine and unfavorable effects in postmenopausal women. Therefore, this study aimed to evaluate the frequently experienced side effects after receiving Sinovac vaccines.

## Materials and methods

This multicenter, cross-sectional study was conducted at multiple hospitals in Karachi, Pakistan. The non-probability sampling method was used. Ethical approval for the study was obtained from Essa General Hospital (Essa/54/2022). The study continued from August 1, 2022, to January 31, 2023, for six months. The study comprised 600 postmenopausal women over the age of 50 years who were vaccinated with two doses of Sinovac COVID-19 vaccination. Menopausal status was defined as any woman who did not have menstrual periods for 12 consecutive months. Premenopausal and perimenopausal women were excluded from the study. The study also excluded anyone who had not received a Sinovac immunization. Participants with ongoing COVID-19 infection were also excluded along with those who were severely immunocompromised or undergoing chemotherapy or radiotherapy for any malignant disease.

Participants’ information was gathered using a proforma (see Appendix 1). Age, gender, comorbidities (including hypertension, diabetes mellitus), vaccine doses, prior COVID-19 infection exposure, and the occurrence of any local and systemic side effects after the first and second doses of the vaccine were all noted as part of their demographic information. Based on their severity, each side effect was categorized as mild, moderate, or severe. The side effects of the vaccine included systemic (i.e., fever, fatigue, chills, headache, myalgia, joint pain, loss of appetite, diarrhea, vomiting, rash, dizziness, cough, dyspnea, and hypersensitivity) and local reactions (i.e., pain, tenderness, swelling, and redness). Based on the recommendations of the Food and Drug Administration, the severity of the side effects was categorized into four groups: (grade 1, mild (awareness of sign or symptom but freely endured); grade 2, moderate (discomfort enough to interfere with typical activities); grade 3, severe (incapacitating with inability to work or do usual activity); grade 4, potentially life-threatening) [14].

Data were entered and analyzed using SPSS Statistics for Windows, Version 26.0 (IBM Corp., Armonk, NY, USA). Demographic parameters, such as gender, comorbidities, and systemic and local side effects in postmenopausal women, were documented as frequencies and percentages. Age, weight, and duration of comorbidities are expressed as means and standard deviations.

## Results

The study included 600 postmenopausal women, with a mean age of 63.93±8.24 years. The postmenopausal women who received the vaccine had an average weight of 63.62 ± 13.87 Kg. The postmenopausal women who received the vaccine had an average height of 5.06 ± 0.70 feet. There were related comorbidities with hypertension and diabetes mellitus in 181 (30.2%) and 40 (6.7%) women, respectively. None of the subjects had a past history of COVID-19, as shown in Table 1.

**Table 1:** Demographic characteristics of post-menopausal women included in the study (n=600) COVID-19: Coronavirus disease 2019

Variable		Mean±SD/n(%)
Age (years)		63.93±8.24
Weight (kg)		63.62±13.87
Height (feet)		5.06±0.70
Hypertension, duration (years)		3.54±0.49
Diabetes mellitus, duration (years)		3.00±2.02
Gender	Female	600(100.0%)
Hypertension	Yes	181(30.2%)
	No	419(69.8%)
Diabetes mellitus	Yes	40(6.7%)
	No	560(93.3%)
Previous COVID-19 infection	Yes	0(0.0%)
	No	600(100.0%)

Following the first dose of the vaccine, various side effects were reported by the participants. Fever was the most often reported side effect, with 349 (58.2%) participants reporting it and 198 (56.7%) participants reporting it as mild. There was pain (208, 34.7%) and swelling at the injection site (190, 31.7%), with the pain being reported as of a mild intensity (190, 91.3%) and the swelling at the injection site being reported as of a moderate intensity (134, 70.5%). Injection site burning was another significant side effect mentioned by 170 (28%) participants; however, 152 (89.4%) said it was rather minor. Furthermore, 130 (21.7%) participants reported chills, 112 (18.7%) reported rashes, 112 (18.7%) expressed fatigue, 92 (15.3%) reported a sore throat, and 76 (12.7%) reported joint pain. Most side effects were mild in intensity. In addition, injection site redness, headache, nausea, flu-like illness, lymphadenopathy, difficulty in breathing, diarrhea, and chest pain were observed to be of low frequency and mild intensity, as shown in Table 2.

**Table 2:** The side effects distribution and severity level in postmenopausal women after receiving the first dose of Sinovac vaccine

Variable	Vaccine side effect		Severity of side effect		
	Present, n(%)	Absent, n(%)	Mild, n(%)	Moderate, n(%)	Severe, n(%)
Pain at the site of injection	208(34.7%)	392(65.3%)	190(91.3%)	18(8.7%)	0(0.0%)
Swelling at the site of injection	190(31.7%)	410(68.3%)	56(29.5%)	134(70.5%)	0(0.0%)
Redness at the site of injection	74(12.3%)	526(87.7%)	74(100.0%)	0(0.0%)	0(0.0%)
Lymphadenopathy	20(3.3%)	580(96.7%)	0(0.0%)	20(100.0%)	0(0.0%)
Fever (temperature >37.8 ˚C)	349(58.2%)	251(41.8%)	198(56.7%)	113(32.4%)	38(10.9%)
Headache	77(12.8%)	523(87.2%)	19(24.7%)	58(75.3%)	0(0.0%)
Nausea	78(13.0%)	522(87.0%)	60(76.9%)	18(23.1%)	0(0.0%)
Rashes	112(18.7%)	488(81.3%)	74(66.1%)	20(17.9%)	18(16.1%)
Burning at injection site	170(28.3%)	430(71.7%)	152(89.4%)	18(10.6%)	0(0.0%)
Flu-like illness	76(12.7%)	524(87.3%)	18(23.7%)	58(76.3%)	0(0.0%)
Anxiety	56(9.3%)	544(90.7%)	56(100.0%)	0(0.0%)	0(0.0%)
Myalgia	94(15.7%)	506(84.3%)	74(78.7%)	0(0.0%)	20(21.3%)
Fatigue	112(18.7%)	488(81.3%)	54(48.2%)	58(51.8%)	0(0.0%)
Joint pain	76(12.7%)	524(87.3%)	76(100.0%)	0(0.0%)	0(0.0%)
Chills	130(21.7%)	470(78.3%)	110(84.6%)	20(15.4%)	0(0.0%)
Cough	78(13.0%)	522(87.0%)	40(51.3%)	20(25.6%)	18(23.1%)
Sore throat	92(15.3%)	508(84.7%)	92(100.0%)	0(0.0%)	0(0.0%)
Shortness of breath	78(13.0%)	522(87.0%)	78(100.0%)	0(0.0%)	0(0.0%)
Diarrhea	74(12.3%)	526(87.7%)	56(75.7%)	18(24.3%)	0(0.0%)
Chest pain	94(15.7%)	506(84.3%)	38(40.4%)	56(59.6%)	0(0.0%)

It was discovered that the side effects after receiving the second dose of vaccination were essentially identical to those following the first dose; however, they differed significantly in frequency and severity. After the second dose, 234 (39.5%) participants reported fever as their most frequent adverse effect, and 158 (67.5%) of them said it was mild. Burning at the injection site was noted by 136 (22.7%) participants; the majority of them, 116 (85.3%), felt it was moderate in severity. A total of 134 (22.3%) and 113 (18.8%) participants reported injection site pain and swelling, respectively. In terms of severity, it was mild, that is, 116 (86.6%) and 73 (55.7%). Additionally, 132 (22.0%) participants experienced fatigue, whereas 136 (22.7%) participants reported burning at the injection site. Approximately 114 (19.0%) participants reported having rashes, 114 (19.0%) participants reported having chills, and 112 (18.7%) participants reported having lymphadenopathy. Other minor side effects, including injection site redness, flu-like illness, anxiety, joint discomfort, cough, sore throat, diarrhea, and chest pain, were also occasionally noticed. It is interesting to note that none of the women experienced nausea, as shown in Table 3.

**Table 3:** The side effects distribution and severity level in postmenopausal women after receiving the second dose of Sinovac vaccine

Variable	Vaccine side effect		Severity of side effect		
	Present, n(%)	Absent, n(%)	Mild, n(%)	Moderate, n(%)	Severe, n(%)
Pain at the site of injection	134(22.3%)	466(77.7%)	116(86.6%)	18(13.4%)	0(0.0%)
Swelling at the site of injection	113(18.8%)	487(81.2%)	73(55.7%)	58(44.3%)	0(0.0%)
Redness at the site of injection	36(6.0%)	564(94.0%)	36(100.0%)	0(0.0%)	0(0.0%)
Lymphadenopathy	112(18.7%)	488(81.3%)	94(83.9%)	18(16.1%)	0(0.0%)
Fever (temperature >37.8 ˚C)	234(39.0%)	366(61.0%)	158(67.5%)	36(15.4%)	0(0.0%)
Headache	94(15.7%)	506(84.3%)	58(61.7%)	36(38.3%)	0(0.0%)
Nausea	0(0.0%)	600(100.0%)	0(0.0%)	0(0.0%)	0(0.0%)
Rashes	114(19.0%)	486(81.0%)	94(82.5%)	0(0.0%)	20(17.5%)
Burning at injection site	136(22.7%)	464(77.3%)	116(85.3%)	20(14.7%)	0(0.0%)
Flu-like illness	36(6.0%)	564(94.0%)	36(100.0%)	0(0.0%)	0(0.0%)
Anxiety	56(9.3%)	544(90.7%)	56(100.0%)	0(0.0%)	0(0.0%)
Myalgia	112(18.7%)	488(81.3%)	94(83.9%)	18(16.1%)	0(0.0%)
Fatigue	132(22.0%)	468(78.0%)	74(56.1%)	58(43.9%)	0(0.0%)
Joint pain	58(9.7%)	542(90.3%)	40(69.0%)	0(0.0%)	18(31.0%)
Chills	114(19.0%)	486(81.0%)	36(31.6%)	78(68.4%)	0(0.0%)
Cough	18(3.0%)	582(97.0%)	18(100.0%)	0(0.0%)	0(0.0%)
Sore throat	56(9.3%)	544(90.7%)	56(100.0%)	0(0.0%)	0(0.0%)
Shortness of breath	98(16.3%)	502(83.7%)	78(79.6%)	20(20.4%)	0(0.0%)
Diarrhea	18(3.0%)	582(97.0%)	0(0.0%)	18(100.0%)	0(0.0%)
Chest pain	96(16.0%)	504(84.0%)	18(18.8%)	78(81.3%)	0(0.0%)

## Discussion

In this study, we found that adverse reactions of the Sinovac COVID-19 vaccine were mild to moderate in postmenopausal women, and the commonest adverse reactions included fever, and pain and swelling at the injection site. It is vital to offer a safe and effective COVID-19 vaccination and anticipate its adverse effects to counter the devastating consequences of the COVID-19 endemic on humans. Thus, the Sinovac vaccine’s local and systemic side effects in postmenopausal women were demonstrated in this study.

In a cross-sectional study, 780 Turkish medical experts who had received the CoronaVac vaccine participated. Approximately four weeks after vaccination, both general and local side effects started to appear. Approximately 62.5% of the 780 participants reported a minimum of one side effect. The most frequent pain at the injection site was reported by 41.5% of participants, whereas tiredness (23.6%), headaches (18.7%), myalgia (11.2%), and pain in joints (5.9%) were the most common general adverse events (SEs). Additionally, female medical professionals suffered considerably more from general and local side effects [15]. Our study, which contrasted with the previously mentioned research, revealed 349 (58.2%) participants, of whom 198 (56.7%) reported mild fever after receiving the first dose, and 234 (39.0%) recipients, after receiving the second dose.

Similarly, another observational cross-sectional study in Brazil’s early stages of the vaccination campaign revealed the side effects of Sinovac vaccines in pregnant and postpartum women. Systemic side effects were the most common reported (82.07%), followed by local (11.93%) and maternal (4.74%), with the majority of these events being categorized as non-severe (90.65%) [16]. The present study supported the findings of other studies and found that after receiving the first and second doses, respectively, 349 (58.2%) and 234 (39.0%) individuals reported experiencing a systemic adverse effect such as fever. In addition, there were noticeable local side effects at the injection site, including pain, swelling, and burning. In terms of severity, 38 (10.9%) participants experienced high-grade fever after receiving the first dose, although most side effects were mild in nature.

Likewise, a survey was structured to obtain information from 6,115 Brazilian healthcare professionals with the predominant age group being 30-39 years (31.3%), 67.3% of participants were women, 73.2% of them were doctors, and nearly half provided front-line treatment for COVID-19. Approximately two-thirds of them received the CoronaVac vaccine, and approximately 60% experienced some negative effects. However, minor side effects, such as headache, tiredness, and injection site pain, were more common [17]. However, Kaya and Pirincci [18], in their prospective study of 329 health experts who were vaccinated with the CoronaVac vaccine in Turkey, reported pain, swelling, headache, fever, redness, tiredness, nausea, allergic reactions, itching, and myalgia in only 33.2% of participants. Headache, sleepiness or lethargy, discomfort, and redness were the three most frequent severe adverse effects.

This multicenter, cross-sectional study included 800 participants who had received complete Sinovac vaccination. Of the 800 participants, 534 (66.8%) were men and 266 (33.3%) were women, with a mean age of 41.20 ± 13.70 years. Fever was the most frequently reported adverse reaction in 350 (43.8%) participants after receiving the first dose of Sinovac vaccination. Other frequent side effects included soreness and injection site swelling, which were experienced by 238 (29.8%) participants and 228 (28.5%) recipients. Fever was the most frequent adverse reaction following the second Sinovac dose, as reported by 262 (32.8%) subjects. Sinovac was tolerated at both dosages, and most side effects were mild and self-limiting [19]. These findings were similar to those of the above-cited research and indicated that the most commonly reported side effect was fever after receiving the first and second doses, followed by injection site pain and swelling.

According to studies, individuals’ post-vaccination high fevers were related to the type of vaccine [20,21]. In addition, it has been emphasized that following the second dose, high fever is more common [22]. According to studies, many disorders slow down the COVID-19 infection’s ability to regress [22]. Diabetes [23], hypertension [24], and chronic obstructive pulmonary disease [25] are a few of these illnesses. Studies on drug usage showed that people who took oral contraceptives, antihistamines, and antihypertensive drugs experienced different side effects (local and systemic adverse effects increased in people who frequently took medication owing to their illnesses) [26]. There was no correlation between vaccine adverse effects and medicine, according to a different study, which found that people with chronic diseases were more susceptible to them [27]. These findings were not corroborated with our study’s findings and revealed that hypertension and diabetes mellitus were found to be present as associated comorbidities in 181 (30.2%) and 40 (6.7%) women, respectively, showing no connection with the presence of side effects with the Sinovac vaccine.

Another descriptive study included 355 nurses who were vaccinated with the Sinovac COVID-19 vaccine in Turkey. Of these, 82.3% were female. Their mean BMI was 24.87±4.54 and their median age was 35.42 ± 9.67 years. Pain was the most frequent local adverse effect following immunization reported by 54.6% of the participants, and tiredness (39.2%) and headache (34.1%) were the most frequent general side effects. Nurses who were diagnosed with COVID-19 before receiving the vaccination reported feeling tired more frequently (p=0.004). Nurses with chronic conditions were more likely to experience the systemic side effect of fever [28]. These findings were inconsistent with the present study and showed that the mean age of menopausal women was 63.93 ± 8.24 years, and the most common reported systemic side effect was fever in 349 (58.2%) participants after the first dose. Most participants had a mild fever, whereas 234 (39.0%) participants had a fever after receiving the second dose.

Our study has some limitations. The inability to link the efficiency of the COVID-19 vaccine with the innate resistance that develops after COVID-19 infection is because it has been shown in multiple studies that the natural defense offered by COVID-19 is more protective, long-lasting, and does not have coagulation side effects. Furthermore, the study may not be representative of the entire population as it only involved a few hospitals and vaccination centers in the city. Therefore, more thorough research on the widely available COVID-19 vaccines should be conducted in this area of Pakistan.

## Conclusions

This study concluded that the most commonly reported side effects among postmenopausal women were fever, pain, and swelling at the injection site after receiving both doses of Sinovac vaccine. These overall side effects were generally mild to moderate in intensity, not life-threatening, and did not require hospitalization, although fever was reported in severe intensity in some participants, particularly after the first dose. It is essential to accurately inform the public about vaccination side effects, potential adverse responses, and the safety of administered immunization. However, additional research evaluating a longer period of observation is still required to provide a more comprehensive safety assessment for the immunization administered to these women.

## Appendices

PROFORMA

Name_____________

Gender_____________

Age: ______(Years)

Comorbidities

Hypertension               Yes                  No              Diabetes Mellitus:  Yes                   No

Duration of diabetes ___________________________

Duration of  hypertension___________________________

Family history

Hypertension:              Yes                  No                   Diabetes:         Yes                  No

Duration of diabetes ___________________________

Duration of  hypertension___________________________

Dose

First_________, Second____________, Booster

Side effects of the vaccine were evaluated as per Table 4.

**Table 4:** Proforma for side effects of vaccine

Side Effects	Yes	No	If Yes			
			Mild	Moderate	Severe	Life Threatening
Pain at the site of injection						
Swelling or soreness at the site of injection						
Redness at the site of injection						
Lymphadenopathy						
Fever (temperature above 37.8 ˚C)						
Headache						
Nausea						
Rashes						
Flu						
Anxiety						
Myalgia						
Fatigue						
Joint pain						
Anaphylaxis						
Chills						
Cough						
Swelling of glands						
Sore throat						
Shortness of breath						
Diarrhea						
Chest Pain						
Any serious Adverse Event (AE)						
Specify serious AE if any:						

The severity of side effect was estimated as per Table 5.

**Table 5:** Estimation of symptom severity

Mild	Moderate	Severe	Potentially life-threatening
Symptoms causing no or minimal interference with usual social & functional activities	Symptoms causing greater than minimal interference with usual social & functional activities	Symptoms causing inability to perform usual social & functional activities	Symptoms causing inability to perform basic self-care functions OR Medical or operative intervention indicated to prevent permanent impairment, persistent disability, or death
